# Current review of Excimer laser Trabeculostomy

**DOI:** 10.1186/s40662-020-00190-7

**Published:** 2020-05-05

**Authors:** Georges M. Durr, Marc Töteberg-Harms, Richard Lewis, Antonio Fea, Paola Marolo, Iqbal Ike K. Ahmed

**Affiliations:** 1 Department of Ophthalmology, Université de Montréal, Montreal, Quebec Canada; 2 Department of Ophthalmology, Centre Hospitalier Universitaire de Montréal (CHUM), 1051 Rue Sanguinet, Montreal, H2X 3E4 Quebec Canada; 3 Department of Ophthalmology, University Hospital Zurich, Zurich, Switzerland; 4 Sacramento Eye Consultants, Sacramento, California USA; 5 Struttura Complessa Oculistica, Città della Salute e della Scienza di Torino, Dipartimento di Scienze Chirurgiche, Università degli Studi di Torino, Torino, Italy; 6 Department of Ophthalmology & Vision Sciences, University of Toronto, Toronto, Ontario Canada

**Keywords:** Excimer laser trabeculostomy, Trabeculotomy, ELT, Intraocular pressure, Glaucoma, MIGS, Cataract

## Abstract

**Background:**

Excimer laser trabeculostomy (ELT) is a microinvasive glaucoma surgery (MIGS) that creates multiple laser channels through the trabecular meshwork using a cold laser system, which minimizes tissue fibrosis and aids in bypassing the main area of resistance to aqueous outflow. The purpose of this review is to evaluate the current body of evidence surrounding ELT in terms of efficacy and review the safety profile of the procedure.

**Main text:**

Studies screened had to show clear inclusion and exclusion criteria as well as well-defined outcome measures. PubMed, MEDLINE, EMBASE and the Cochrane Controlled Trial Database were searched. Preferred Reporting Items of Systematic Reviews (PRISMA) guidelines were used to assess for study quality and for any bias. Sixty-four articles were initially identified with 18 meeting preliminary screening criteria. Ultimately, 8 studies met inclusion criteria and 2 additional non-referenced publications were also included: 1 randomized control trial, 4 prospective case series and 5 retrospective studies. Overall studies showed moderate intraocular pressure (IOP) lowering of between 20% and 40% from baseline without medication washout and mostly a decrease in glaucoma medications with few complications.

**Conclusion:**

Current literature shows a significant IOP-lowering effect of ELT with a favorable safety-profile in standalone cases or combined with cataract surgery. Limitations to these studies are the lack of controls and washout IOP. Overall, ELT is an attractive MIGS option that does not require any residual device remaining in the angle.

## Background

The glaucoma surgical landscape has significantly changed in recent years. The conventional treatment paradigm began with hypotensive drops, laser surgery and traditional filtering surgery such as tube shunts and trabeculectomies. Surgical practice patterns in the US now reflect an increase in microinvasive glaucoma surgery (MIGS) as an option for glaucoma management [1]. MIGS is an alternative to traditional glaucoma surgeries predicated on less invasive, more physiologic and less risky approaches to outflow enhancement. As evidenced by the Early Manifest Glaucoma Trial, nearly two thirds of patients initially present with early or moderate glaucoma [2]. As we move to a more interventional mindset for glaucoma care, mild to moderate disease may benefit from a less invasive procedure with modest intraocular pressure (IOP) lowering as this can aid in disease control, improve compliance or decrease eyedrop load.

MIGS aims to bypass the main area of conventional outflow resistance, the trabecular meshwork (TM), through stenting, dilating or cutting procedures. Aqueous humor travels through Schlemm’s canal, traverses the TM and then drains through collector channels followed by aqueous veins and finally, episcleral veins to eventually enter the general circulation. Most aqueous veins are found in the inferior quadrants (more so in the infero-nasal quadrant), and there are generally two to three of these veins in a normal eye. Aqueous humor flow is believed to be controlled by a pump-like mechanism. The cardiac pulse, blinking and eye movements are thought to be the driving forces for transient oscillations in the collector system. The ability of Schlemm’s canal to vary in volume and the TM to distend and recoil are characteristics of the outflow system that allow the pump to push aqueous downstream [3]. MIGS allows surgeons to tap into this mechanism and thereby treat the main area of resistance to outflow.

The chief hurdle for glaucoma procedures, including MIGS, is postoperative scarring, which often leads to a lack of long-term efficacy. What likely differentiates one MIGS procedure from the other is the ability to access the relevant distal outflow vessels, maintain the canal pump mechanism, and minimize postoperative healing and scarring.

Among the different options, excimer laser trabeculostomy (ELT; AIDA, Glautec AG, Nurnberg, Germany) was one of the first procedures to enter the MIGS surgical space with Berlin et al. initially proposing the concept of ELT in the late 1980s [4]. This predecessor device has been supplanted by the ExTra Laser System (MLase AG, Germering, Germany; ExTra ELT), which received a CE mark in 2014.

### What is ELT?

**Fig. 1:**
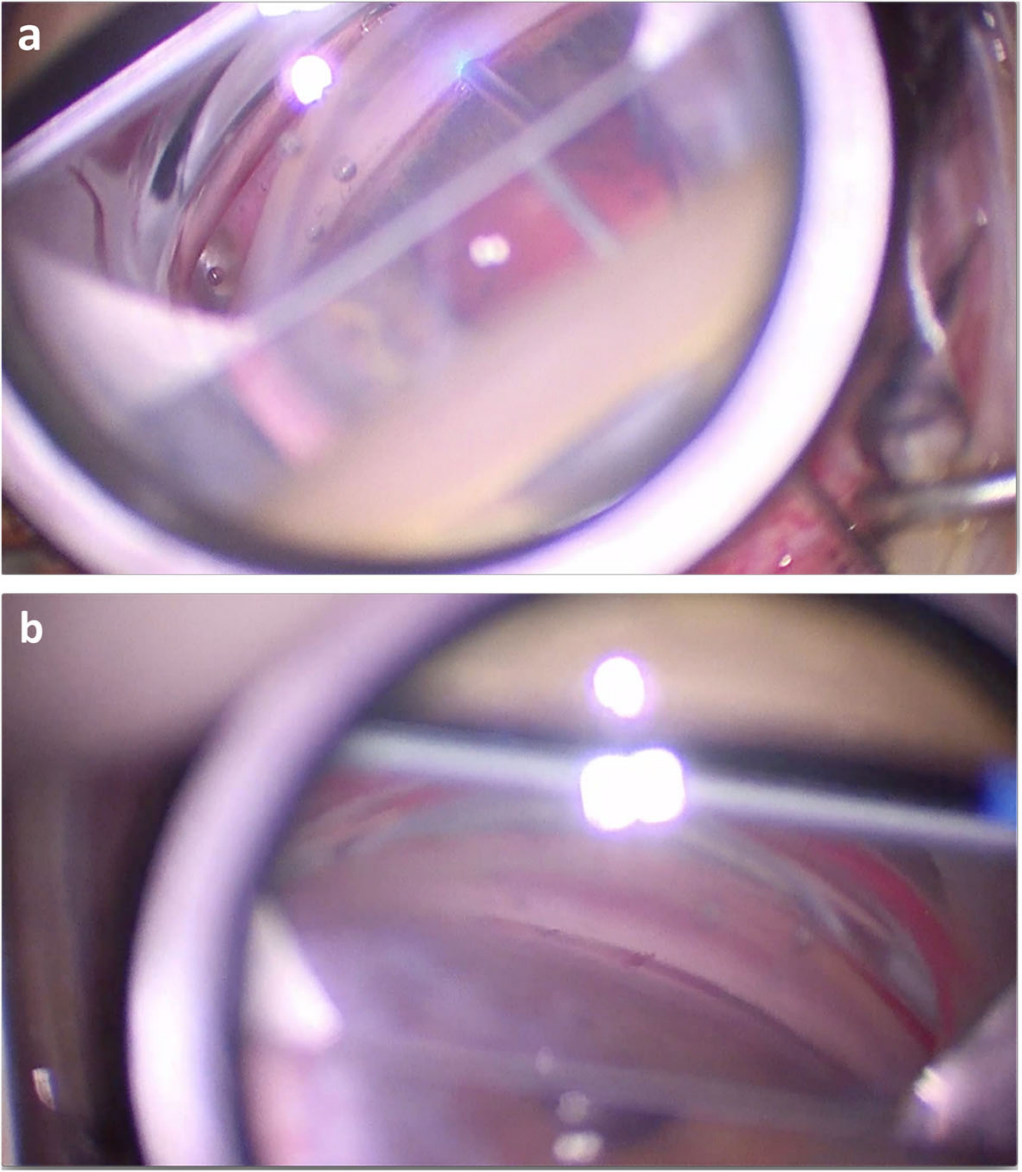
Gonioscopic view of the Excimer Laser Trabeculostomy. View of the probe contacting the trabecular meshwork (**a**) and the subsequent trabeculostomy openings with bubble formation (**b**). Courtesy: Iqbal Ike K. Ahmed

ExTra ELT utilizes excimer laser technology to ablate portions of the TM and inner wall of Schlemm’s canal. Excimer laser technology has been used for decades in refractive surgery because of its great precision, minimal thermal damage, very low tissue penetrance and non-lethality to adjacent cells. The photoablative properties of the laser breaks carbon-nitrogen and carbon-carbon tissue bonds with low energy (4 eV) and does not transmit energy through water, which makes it an attractive option for intraocular use [5]. In refractive surgery, an excimer 193-nm laser is used. However, this wavelength is not transmissible through fiberoptics over a longer distance and can damage adjacent tissue if performed intracamerally [4, 6]. To overcome the problem of being transmissible through fiberoptics, the ExTra Laser System uses a 308-nm xenon chloride (XeCl) excimer laser which creates 200-μm trabeculostomy openings (laser channels) through the TM and inner wall of Schlemm’s canal with an estimated treatment depth of 20 μm (Fig. 1). The short pulse energy applied with Extra ELT is 1.2–1.3 mJ with a duration of 80 ns. The quartz fiber-optic probe is powered by an excimer laser transmitting light to the TM for precise cold photoablation of the tissue, thereby obviating thermal damage [7]. An animal study on 10 rabbit eyes showed that trabecular cell mitochondria and endoplasmic reticula dilate initially as a result of the laser [8]. These cellular changes are transient and resolve spontaneously. A perhaps more compelling study finding was the absence of fibroblast migration up to 5 weeks after the procedure. This theoretically limits the amount of postoperative scarring.

### Surgical procedure

The procedure can be performed standalone or combined with cataract surgery. When combined with phacoemulsification, ELT is typically performed following cataract extraction (CE). The probe with an outer diameter of 500 μm requires a 0.8 mm clear corneal incision. Under gonioscopic or endoscopic visualization, it is advanced bevel up, through the anterior chamber previously filled with viscoelastic and placed in direct contact with the TM. A foot pedal system is used to apply the laser energy. Usually, ten microchannels are created over 90 degrees, 500 μm apart. Blood and microbubbles often seen after the laser is applied are likely the result of blood reflux from Schlemm’s canal. One potential additional effect from the procedure is from the pneumatic canaloplasty [4, 9]. As the photoablated tissue is transformed into gas, it has been hypothesized that these bubbles dilate Schlemm’s canal, pushing the outer wall as well as adjacent collector channels.

After the procedure, patients are instructed to either stop all or continue some of their glaucoma medications as per surgeon preference. Typically, patients are started on combination antibiotic and steroid drops 3 or 4 times a day and tapered over 2–4 weeks.

### Why the renewed buzz around ELT?

Around the time ELT was initially developing and being introduced into the clinical landscape, phacoemulsification was in the beginning stages of adoption, and few were approaching glaucoma therapy through an ab interno approach. At that time, argon laser trabeculoplasty and later, selective laser trabeculoplasty (SLT) were introduced using an incisionless approach with thermal energy to remodel the TM and allow for increased outflow. One drawback of these procedures is the creation of scar tissue as a result of thermal damage [4, 9]. With the popularization of MIGS, multiple ab interno options are now commonly used in combination with cataract surgery or as standalone treatments. ELT returned to the treatment armamentarium with the arrival of a new CE-marked ExTra ELT laser platform in 2014 that is currently only available in the European Union. This platform has several potential advantages: it minimizes thermal damage, limits hyphemas and enables the creation of multiple implant-free microchannels through the TM. Ongoing clinical trials are anticipated to supplement the body of knowledge about this emerging device-free alterative for lowering IOP.

The purpose of this review is to summarize the current body of literature describing ELT and to provide an overview of the results from early experience with the device.

## Main text

### Methods

This literature review was performed in accordance to the Preferred Reporting Items of Systematic Reviews (PRISMA) guidelines [10]. PubMed, MEDLINE, EMBASE and the Cochrane Controlled Trial Database and reference lists of original studies as well as reviews were searched. Keywords used in the search were ‘excimer laser trabeculotomy’ and ‘excimer laser trabeculostomy’. Experts were contacted to identify unpublished trials. The date of the last search was September 4, 2019. No language restrictions were imposed.

Sixty-four articles were screened using a two-stage process. The first screening consisted of a review of titles and abstracts. Preliminary screening eliminated descriptive articles and general reviews that did not contain results of research studies on ELT. For those that passed the first stage, a reading of the full-text was performed. Any article written in a foreign language was translated by a medical translator into English.

The primary outcome of this review was to assess the current body of literature and the efficacy and safety-profile of ELT.

#### Data collection

All studies selected included data describing baseline characteristics (age, sex, ethnicity), eye laterality, cup-to-disc ratio, glaucoma stage (mean deviation on visual field), follow-up time and technique variations. IOP measurements were the primary outcomes compared; means or medians, IOP at defined follow-up intervals and % IOP reduction from baseline were compared. Success or failure criteria were also examined when available. Topical medication use, pre- and postoperatively, were also obtained. Studies were also screened for concomitant cataract surgery at the time of ELT. Intraoperative and postoperative complications and adverse events were also detailed and tallied.

### Results

A total of 64 articles were identified by the literature review. Following initial assessment, 18 articles met the first screening criteria. Upon full text review, 8 articles were included in this review that had defined their methods, results and discussion [9, 11–17] (Table 1). Two additional non-referenced publications also were included in the review.

**Table 1:** Excimer laser trabeculostomy: breakdown of current studies

Authors	Type of study	Inclusion criteria	ELT spots	Study duration	Intervention/comparator	N of eyes	Preop IOP (mmHg)	Preop meds	Postop IOP (mmHg)	Postop meds
*Randomized control trial*										
Babighian et al.	Prospective, comparative	Mild to moderate POAG	8	2 years	ELT	15	25 ± 1.9	2.3 ± 0.7	17.6 ± 2.2	0.7 ± 0.8
					SLT	15	23.9 ± 0.9	2.2 ± 0.7	19.1 ± 1.8	0.9 ± 0.8
*Prospective case series*										
Babighian et al.	Prospective nonrandomized trial, comparative	POAG	8	2 years	ELT	21	24.8 ± 2.0	2.24 ± 0.6	16.9 ± 2.1	0.71 ± 0.8
					Drops	21	21.67 ± 1.6	–	21.0 ± 0.5	–
Töteberg-Harms et al.	Prospective consecutive case series	OHT or mild to moderate OAG	10	1 year	ELT + CE	64	19.8 ± 5.3	2.4 ± 1.1	15.2 ± 4.4	1.5 ± 1.4
Berlin et al.^a^	Prospective interventional case series	OAG	5–10	8 years	ELT	46	22.9 ± 5.4	1.6 ± 0.7	16.1 ± 3.4	1.2 ± 1.2
					ELT + CE	37	25.1 ± 6.1	1.3 ± 0.7	14.2 ± 3.1	1.8 ± 0.8
Kleineberg et al.^a^	Prospective observational study, comparative	Mild to moderate OAG	10	1 year	ELT + steroids	25	24.9 ± 2.7	2.0	17.0 ± 4.0	0.4
					ELT + NSAID	23	26.3 ± 2.5 (*n* = 23)	1.7 (*n* = 23)	16.0 ± 3.5 (*n* = 22)	0.6 (*n* = 22)
					ELT + CE	43	22.2 ± 1.7	1.4	12.0 ± 2.8	0.2
				5 years	ELT + steroids	25	24.9 ± 2.7 (*n* = 25)	2.0 (*n* = 25)	15.0 ± 3.2 (*n* = 15)	0.8 (*n* = 15)
					ELT + NSAID	23	26.3 ± 2.5 (*n* = 23)	1.7 (*n* = 23)	16.0 ± 2.6 (*n* = 15)	0.9 (*n* = 15)
					ELT + CE	43	22.2 ± 1.7 (*n* = 43)	1.4 (*n* = 43)	14.0 ± 2.6 (*n* = 31)	0.9 (*n* = 31)
*Retrospective comparative study*										
Wilmsmeyer et al.	Retrospective case series, comparative	OHT or mild to moderate OAG	10	1 year	ELT	75	24.1 ± 0.7 (*n* = 69)	1.9 ± 0.1 (*n* = 69)	18.8 ± 0.8 (*n* = 37)	1.8 ± 0.2 (*n* = 37)
					ELT + CE	60	22.4 ± 0.6 (*n* = 57)	1.1 ± 0.2 (*n* = 57)	16.4 ± 0.4 (*n* = 35)	1.2 ± 0.2 (*n* = 35)
				2 years	ELT	75	24.1 ± 0.7 (*n* = 69)	1.9 ± 0.1 (*n* = 69)	16.8 ± 1.0 (*n* = 15)	1.5 ± 0.3 (*n* = 15)
					ELT + CE	60	22.4 ± 0.6 (*n* = 57)	1.1 ± 0.2 (*n* = 57)	12.8 ± 1.5 (*n* = 4)	1.8 ± 0.9 (*n* = 4)
Töteberg-Harms et al.	Retrospective interventional case series, comparative	Mild to moderate OAG	10	4 years	ELT + CE	51	19.0 ± 9.0 (*n* = 51) *median ± IQR*	2 ± 1 (*n* = 51)	14.0 ± 5.5 (*n* = 50)	1 ± 2 (*n* = 50)
					Trab+CE	62	22.8 ± 6.3 (*n* = 62)	2 ± 1 (*n* = 62)	14.0 ± 3.5 (*n* = 58)	0 (*n* = 58)
Jozic et al.	Retrospective interventional case series, comparative	OAG	10	1 year	CE	38	16.7 ± 3.8	1.1 ± 0.6	15.2 ± 3.1	1.0 ± 0.7
					ELT + CE	105	17.8 ± 4.3	1.4 ± 0.7	13.2 ± 2.3	0.5 ± 0.8
					ELT + Trabectome	102	19.3 ± 4.6	1.3 ± 0.8	13.8 ± 2.2	0.5 ± 0.7
*Retrospective case series*										
Töteberg-Harms et al.	Retrospective case series	OHT or mild to moderate OAG	10	1 year	ELT + CE	28	25.3 ± 2.9	2.3 ± 1.3	16.5 ± 5.0	1.5 ± 1.4
Moreno Valladares et al.	Retrospective interventional case series	Mild to moderate OAG	10	6 months	ELT or ELT + CE	27	21.2	1.8	17.8	0.7

^a^Unpublished studies

*preop*= preoperative; *IOP*= intraocular pressure; *postop*= postoperative; *SD*= standard deviation; *IQR*= interquartile range; *POAG*= primary open-angle glaucoma; *OAG*= open-angle glaucoma; *OHT*= ocular hypertension; *NSAID*= non-steroidal anti-inflammatory drug; *ELT*= Excimer laser trabeculostomy; *CE*= cataract Extraction; *Trab*= trabeculectomy.

#### Randomized control trial

The highest level of evidence was provided by Babighian et al. with a single center randomized control trial in Italy comparing ELT (*n* = 15) with 180-degree SLT (*n* = 15) over a 2-year follow-up in mild to moderate primary open angle glaucoma (POAG) [11]. All ELT and SLT were performed by the same operator (8 spots with ELT and 50 spots with SLT using 0.7–1.0 mJ of energy) and post-operative visits were performed by a masked physician. Complete success was defined as a ≥ 20% reduction in IOP without glaucoma medications (meds) and qualified success as ≥20% reduction in IOP with meds. Complete success was obtained for 53% (8/15) of ELT eyes compared to 40% (6/15) of SLT eyes (*p* = 0.35, Fisher’s exact test). Regarding qualified success, 87% (13/15) of ELT eyes succeeded compared to 67% (10/15) of SLT eyes (*p* = 0.5, Fisher’s exact test). At 2 years, ELT decreased mean IOP from 25.0 ± 1.9 mmHg to 17.6 ± 2.2 mmHg (30% reduction; *p* < 0.0001) and SLT decreased mean IOP from 23.9 ± 0.9 mmHg to 19.1 ± 1.8 mmHg (21% reduction, p < 0.0001) with similar meds reduction compared to baseline [ELT: 2.3 ± 0.7 to 0.7 ± 0.8 (*p* = 0.005) vs SLT: 2.2 ± 0.7 to 0.9 ± 0.8 (p < 0.0001)]. Complications included transient IOP spikes in both groups (3/15 with ELT and 2/15 with SLT) and mild reflux bleeding in 12/15 ELT eyes that resolved within 5 days.

#### Prospective case series

A prospective single-center case series by Babighian et al. examined results of ELT at 2-year follow-up in POAG with contralateral eye as a control [13]. ELT was performed over 90 degrees with 8 spots. Complete success was defined as a ≥ 20% reduction in IOP without any meds and qualified success as ≥20% reduction in IOP with meds. Mean IOP in the ELT group at baseline was 24.8 ± 2.0 mmHg and decreased to 16.9 ± 2.1 mmHg (32% reduction, *p* < 0.0001) at 2 years. Controls had no significant change to IOP at baseline 21.67 ± 1.6 mmHg and 21.0 ± 0.5 mmHg at 2 years. Complete success was obtained in 52% of eyes and qualified success in 90% of eyes. The only complications reported were small hyphemas in 80% of patients that spontaneously resolved within 5 days.

Töteberg-Harms et al. performed a 1-year prospective case series on 64 consecutive eyes undergoing combined ELT + CE in patients with open angle glaucoma (OAG) [15]. ELT was performed over 90 degrees with 10 spots. Two groups were created based on preoperative IOP with group 1 having IOP ≤21 mmHg and group 2 having IOP > 21 mmHg. Success was defined as IOP ≤21 mmHg and ≥ 20% IOP reduction with same or less meds. Overall IOP dropped from 19.8 ± 5.3 mmHg to 15.2 ± 4.4 mmHg at 1 year (23% reduction): group 1 IOP fell from 16.5 ± 2.9 mmHg to 14.6 ± 3.7 mmHg (12% reduction) and group 2 IOP from 25.8 ± 2.9 mmHg to 16.4 ± 5.4 mmHg (37% reduction). Medication reduction was similar among groups (group 1: 2.5 ± 1.0 to 1.4 ± 1.3 at 1 year; group 2: 2.2 ± 1.4 to 1.6 ± 1.5 at 1 year). Overall success was obtained in 47% of the sample with a success rate of 38% in group 1 and 63% in group 2. Seven patients (11%) required further glaucoma surgery and a few patients had mild anterior chamber reaction. There was no mention of hyphema rates or IOP spikes.

#### Retrospective comparative studies

Lozic et al. reviewed a 1-year retrospective interventional case series comparing CE with ELT + CE and Trabectome (NeoMedix, Tustin, CA, USA) + CE in patients with OAG [17]. ELT was performed over 90 degrees with 10 spots and Trabectome was performed over 90 to 120 degrees. Mean IOP at baseline for the different groups were 16.7 ± 3.8 mmHg on 1.1 ± 0.6 meds (CE group, *n* = 38), 17.8 ± 4.3 mmHg on 1.4 ± 0.7 meds (ELT + CE group, *n* = 105) and 19.3 ± 4.6 mmHg on 1.3 ± 0.8 meds (Trabectome+CE group, *n* = 102). At 1 year, mean IOP and meds dropped to 15.2 ± 3.1 mmHg on 1.0 ± 0.7 meds (CE group), 13.2 ± 2.3 mmHg on 0.5 ± 0.8 meds (ELT + CE group) and 13.8 ± 2.2 mmHg on 0.5 ± 0.7 meds (Trabectome+CE group). Failure was defined as IOP > 21 mmHg or < 20% IOP reduction or hypotony (< 5 mmHg) or loss of light perception vision. Kaplan-Meier curve showed improved mean survival time for the ELT + CE group (20.6 ± 1.0 months) compared to the CE group (13.2 ± 0.4 months) and the Trabectome+CE group (12.9 ± 0.6 months). There were no significant hyphemas or IOP spikes.

Töteberg-Harms et al. performed a 4-year retrospective interventional case series comparing ELT + CE and trabeculectomy (Trab) + CE in patients with OAG [16]. ELT was performed over 90 degrees with 10 spots. Complete success was defined as IOP ≤21 mmHg and ≥ 20% IOP reduction without meds and qualified success as IOP ≤21 mmHg and ≥ 20% reduction in IOP with meds. IOP in the ELT + CE group (*n* = 51) at baseline was a median ± interquartile range (IQR) of 19.0 ± 9.0 mmHg on 2 ± 1 meds; IOP decreased to 15.0 ± 5.0 mmHg on 1 ± 2 meds at 1 year and decreased further to 14.0 ± 5.5 mmHg on 1 ± 2 meds at 4 years. In comparison, baseline IOP in the Trab+CE group (*n* = 62) was 22.8 ± 6.3 mmHg on 2 ± 1 meds; IOP decreased to 13.0 ± 4.5 mmHg at 1 year on 0 meds and to 14.0 ± 3.5 mmHg on 0 meds at 4 years. Loss to follow-up was not reported. Complete success rate in the ELT + CE group was 18% at 1 year and 9% at 4 years; in the Trab+CE group, it was 90% at 1 year and 75% at 4 years. Qualified success rate in the ELT + CE group was 47% at 1 year and 34% at 4 years; in the Trab+CE group, it was 95% at 1 year and 89% at 4 years. Complications were reported as IOP spikes in 10% of the ELT + CE group and hypotony in 22% of Trab+CE group. There was no mention of hyphema rates.

Wilmsmeyer et al. published a 2-year retrospective comparative case series in Germany of ELT (*n* = 75) and ELT combined with CE (*n* = 60) in OAG [12]. ELT was performed over 90 degrees with 10 spots. Success was defined as IOP ≤21 mmHg and ≥ 20% IOP reduction (criteria 1) and as IOP < 18 mmHg and > 30% IOP reduction (criteria 2). IOP for standalone ELT was 24.1 ± 0.7 mmHg at baseline (*n* = 69) followed by 18.8 ± 0.4 mmHg (*n* = 66) at 3 months, 20.0 ± 0.5 (*n* = 51) at 6 months, 18.8 ± 0.8 mmHg (*n* = 37, 22% reduction) at 12 months and 16.8 ± 1.0 mmHg (*n* = 15, 30% reduction) at 24 months (*p* < 0.001 for all). Mean IOP for combined ELT + CE was 22.4 ± 0.6 mmHg (*n* = 57) at baseline, which decreased to 16.5 ± 0.4 mmHg (*n* = 52) at 3 months, 16.1 ± 0.5 mmHg (*n* = 40) at 6 months, 16.4 ± 0.4 mmHg (*n* = 35, 27% reduction) at 12 months and 12.8 ± 1.5 mmHg (n = 4, 47% reduction) at 24 months (*p* < 0.01 for all). Medications remained similar in the ELT and ELT + CE groups (ELT baseline: 1.9 ± 0.1, 12 months: 1.8 ± 0.2 and 24 months: 1.5 ± 0.3; ELT + CE baseline: 1.1 ± 0.2, 12 months: 1.2 ± 0.2 and 24 months: 1.8 ± 0.9). Success by criteria 1 was obtained in 46 and 66% at 12 months in the ELT and ELT + CE groups, respectively. Success by criteria 2 was obtained in 27 and 42% at 12 months in the ELT and ELT + CE groups, respectively. Complications were rare in this series: 2 patients had iris adhesions to the corneal tunnel, 3 had fibrinous anterior chamber reaction (ELT + CE) and 1 patient had a central retinal vein occlusion, 5 months after the ELT + CE. There was no mention of hyphema rates or IOP spikes.

#### Retrospective case series

Töteberg-Harms et al. performed a 1-year retrospective case series in Switzerland on 24 consecutive eyes undergoing combined ELT + CE in patients with OAG [14]. ELT was performed over 90 degrees with 10 spots. Mean IOP at baseline was 25.3 ± 2.9 mmHg and decreased to 16.5 ± 5.0 mmHg at 1 year (IOP reduction 8.8 ± 5.3 mmHg, 37.7%), and medications decreased from 2.3 ± 1.3 mmHg to 1.5 ± 1.4 mmHg, respectively. Four patients required a secondary surgery due to uncontrolled IOP. There was no mention of hyphema rates or IOP spikes.

Moreno Valladares presented a 6-month retrospective interventional case series of his early experience in Spain of ELT and ELT + CE in 27 eyes with OAG [9]. Mean IOP in the ELT group was 21.2 mmHg at baseline on 1.8 meds and decreased to 17.8 mmHg on 0.7 meds at 6 months.

#### Unpublished studies

Two as yet unpublished studies are also noteworthy. Berlin et al. reported on 8-year results of a prospective nonrandomized trial with ELT (*n* = 46) and ELT + CE (*n* = 37) in patients with OAG [18]. Mean IOP decreased 29.7% from 22.9 ± 5.4 mmHg at baseline to 16.1 ± 3.4 mmHg at 8 years in the ELT group and fell 43.4% in the ELT + CE group from baseline 25.1 ± 6.1 mmHg on 1.3 ± 0.7 meds to 14.2 ± 3.1 mmHg on 1.8 ± 0.8 meds.

Kleineberg et al. performed a prospective, observational 60-month single-surgeon study in patients with OAG and no washout period, with three arms: ELT plus post-operative steroid drops (group 1, *n* = 25), ELT plus post-operative nonsteroidal anti-inflammatory drops (NSAIDs) (group 2, *n* = 23) and ELT plus CE (group 3, *n* = 43). ELT was performed with 10 spots. Mean preoperative medicated IOPs were 24.9 ± 6.6 mmHg, 26.3 ± 5.8 mmHg and 22.2 ± 5.5 mmHg, respectively, and decreased at 1 year to 16.3 ± 4.0 mmHg, 15.7 ± 3.5 mmHg and 12.5 ± 2.8 mmHg, respectively. At 60 months, mean IOPs were 15.5 ± 3.2 mmHg (*n* = 15), 16.3 ± 2.6 mmHg (*n* = 15) and 14.1 ± 2.6 mmHg (*n* = 32), i.e., decrements of 37.8, 38 and 36.5%, respectively. Medications changed from 2.04 (baseline, *n* = 25) to 0.44 (1 year, *n* = 25) to 0.8 (5 years, *n* = 15) for group 1; from 1.74 (baseline, *n* = 23) to 0.59 (1 year, *n* = 23) to 0.93 (5 years, *n* = 15) for group 2; and from 1.40 (baseline, *n* = 43) to 0.23 (1 year, *n* = 43) to 0.94 (5 years, *n* = 31) for group 3. No hypotony was observed in any of the groups. Insufficient pressure reduction necessitated secondary surgical interventions in 3/91 patients (3.3%) with 1 patient from group 1 and 2 patients from group 3. Only 3 patients had hyphema > 1 mm and all were self-limited. Only 1 patient had an IOP spike of ≥10 mmHg, which resolved spontaneously by the next follow-up without any intervention. Visual acuity remained the same in groups 1 and 2 while it improved in group 3, which was mainly the result of the CE.

### Discussion

This review allows a comprehensive assessment of the current body of literature describing the use of ELT. All studies have shown moderate IOP lowering at variable time points, between 20 and 40% reduction from baseline without medication washout, and mostly a decrease in glaucoma medications with few complications [9, 11–18]. Although they are not direct comparisons, these results appear comparable or superior to those obtained with other MIGS [19].

Excimer lasers differ from frequency-doubling Yttrium Aluminum Garnet (YAG) lasers in several regards. XeCl lasers emit in the ultraviolet range (308 nm) in contrast to YAG lasers, which emit in the infrared range (1064 nm). Tissue interactions differ following exposure to these divergent wavelengths. With ophthalmic Nd:YAG lasers, the protein-absorbed energy causes thermal coagulation, tissue shrinkage and tissue photodisruption by shock wave. It has been hypothesized that the XeCl laser directly and rapidly breaks protein bonds within cells whereas lasers in the infrared range can only indirectly re-shape tissue through photothermal and photomechanical changes to pigmented cells of the TM. Excimer lasers (excited dimer), using intense UV light, break molecular bonds and liberate molecular fragments while sparing adjacent tissues. One of the advantages of the excimer laser is the short depth of penetration of the laser (30 to 50 μm) [20]. Another differentiating concept is the formation of a plume with excimer lasers, which minimizes tissue damage. After the vapor bubble expands and collapses at the time of a pulse, a plume is created and carries away excess heat, effecting so-called cold ablation. Plumes are created when the laser pulse is shorter than the time required for heat to diffuse out of the irradiated zone. Excimer laser energy is not absorbed by liquids compared to higher wavelength lasers that rely on water absorption and result in higher collateral damage [21]. Considering the need to prevent postoperative healing and scarring to obtain long-term efficacy, the many unique characteristics of excimer lasers make it an attractive alternative for the creation of trabeculostomies.

Some studies have compared the effect of ELT as a standalone intervention or combined with cataract surgery [12, 18]. There may be an additional benefit of combining ELT and cataract surgery as is believed to be the case in other MIGS procedures [22]. Cataract surgery may help potentiate the effect of ELT. The combined or synergistic effect of the two procedures has not yet been fully elucidated because these studies are limited by the lack of control arms. Cataract surgery has a well-documented albeit modest IOP-lowering effect, which may confound the ELT portion of the IOP decrease [23–26]. Ideally, a trial comparing cataract surgery alone or combined with ELT is warranted to better ascertain the additive or synergistic effect from the laser procedure. Another consistent finding was that higher preoperative IOP led to a better response to ELT [15]. This is often the case in most glaucoma trials. When the IOP is lower, achieving success becomes more challenging as a significant drop in IOP is much more difficult to achieve with lower preoperative pressures. Notably, none of the studies in this review used washout or decision IOP (IOP at the time the decision was made to proceed with surgery) values to obtain pre- or post-operative values, which potentially would have provided greater absolute and percent IOP reductions.

The best evidence to date is from an randomized control trial comparing ELT to 180-degree SLT [11]. This was a well-designed study with clear outcome measures and good follow-up, despite a small sample size. ELT appeared to outperform SLT although this was not statistically significant likely due to an underpowered study. Some limitations of this study arose from the study design. SLT was performed over 180 degrees. Arguably, there may be a difference in the treatment effect of 180 degrees compared to 360 degrees; however, some studies have shown equivalent IOP response between 180 and 360 degrees with fewer IOP spikes [27, 28]. Another question that this study raises is whether SLT is the best comparator. SLT is an incision-less procedure using thermal energy to reshape the TM by incompletely understood mechanisms to allow further outflow. Although ELT acts on TM through a cold laser system with less potential for damage, it does require an incision and thus carries a small increased risk of infection compared to SLT. No cases of endophthalmitis were reported in the studies described in this review. Furthermore, ELT must be performed in the operating room (OR) with increased preparation/surgical time compared to SLT, which is performed in the office. When combined with CE, ELT requires additional surgical time [15]. The use of an endoscope or intraoperative gonioscopy to visualize the angle is another potential limitation of the procedure. Surgical skill in the use of an endoscope or with intraoperative gonioscopy to visualize the angle is another potential limitation of the procedure.

Other comparators are trabecular bypass MIGS or trabeculotomies. The potential advantage of ELT over any trabecular bypass device is that ELT does not require any material to remain in the angle with the attendant potential risks of corneal decompensation or device migration. Compared to trabeculotomies, ELT spots are small (200 μm) with lower potential risk of the severe hyphemas that have been reported with trabeculotomies [29–31]. Furthermore, excimer-created openings theoretically would be less likely to scar and/or close compared to traumatic tearing or cutting procedures in the TM. A single retrospective study compared ELT + CE to Trabectome+CE and showed comparable mean IOP and decreased medication use at 1-year [17]. The results for the ELT + CE group were superior to those of the other groups in terms of survival analysis although this finding may have been subject to bias related to nonrandomization or differential preoperative IOPs. To date, no head-to-head prospective randomized study comparing standalone ELT to trabecular bypass MIGS or trabeculotomies has been conducted. One study compared ELT + CE with trabeculectomy+CE with the latter outperforming ELT + CE although the ELT + CE group had a faster recovery and a favorable safety profile (IOP spikes in 10% of the ELT + CE group; hypotony in 22% of Trab+CE group) [16]. The result is not surprising considering that blebs are expected to lower IOP more substantially than a canal-based procedure, which is limited (and protected) by episcleral venous resistance. Trabeculectomy, with both short- and long-term potential bleb complications (hypotony, infection, bleb leaks, choroidal detachments) is typically reserved for more advanced or uncontrolled glaucomas. Trabeculectomy remains the gold standard for glaucoma management worldwide. However, in view of the significant risks, patients and physicians are inclined to choose a bleb-less procedure with modest IOP response for mild or moderate glaucomatous disease. Such procedures are considered adequate to prevent future glaucoma damage by better controlling IOP, decreasing drop load and improving compliance.

Complications were rare in the studies reviewed, and the few reported were mainly due to hyphemas and IOP spikes. The transient perioperative hyphemas were small, unassociated with IOP spikes, did not require treatment and resolved within a few days. Intraoperatively, creation of the laser microchannels produces a small amount of hemorrhage into the anterior chamber, subsequently cleared with irrigation. In effect, this observation confirms the patency of the trabeculostomy and results from blood reflux. To date no study has reported a significant hyphema that lead to uncontrolled IOP or the need for an anterior chamber washout. IOP spikes have been reported in 10–15% of cases in 2 studies and all resolved without the need for further surgical intervention [11, 16]. IOP spikes in these settings may be due to pigment release, inflammation, blood or retained viscoelastic at the end of the procedure.

## Conclusions

Overall, current available evidence show an IOP-lowering effect from ELT alone or in combination with cataract surgery with encouraging results across different studies and patient populations, notably without washout IOP, and a favorable safety profile. Multiple studies, albeit with small sample sizes and variable loss to follow-up, have shown a long-lasting response up to 8 years after the initial surgery. The potential advantages of this procedure are less scarring than results from traditional thermal lasers, repeatability in different quadrants, ease of use, no device left in the angle and, with lower hyphema risks compared to ablative procedures, potentially less secondary synechia to the angle. The procedure also appears to have a favorable safety profile with few intraoperative or postoperative risks. Like any new technology, more studies are needed to better characterize ELT and further substantiate these promising results.

## Data Availability

Data sharing is not applicable to this article as no datasets were generated or analyzed during the current study.
